# How do the rotavirus NSP4 and bacterial enterotoxins lead differently to diarrhea?

**DOI:** 10.1186/1743-422X-4-31

**Published:** 2007-03-21

**Authors:** Mathie Lorrot, Monique Vasseur

**Affiliations:** 1 Hôpital Robert Debré, Service de Pédiatrie Générale, Paris, F-75019, France; 2 INSERM, UMR-S756, Université Paris-Sud 11, Faculté de Pharmacie, Châtenay-Malabry, F-92296, France

## Abstract

Rotavirus is the major cause of infantile gastroenteritis and each year causes 611 000 deaths worldwide. The virus infects the mature enterocytes of the villus tip of the small intestine and induces a watery diarrhea. Diarrhea can occur with no visible tissue damage and, conversely, the histological lesions can be asymptomatic. Rotavirus impairs activities of intestinal disaccharidases and Na+-solute symports coupled with water transport. Maldigestion of carbohydrates and their accumulation in the intestinal lumen as well as malabsorption of nutrients and a concomitant inhibition of water reabsorption can lead to a malabsorption component of diarrhea. Since the discovery of the NSP4 enterotoxin, diverse hypotheses have been proposed in favor of an additional secretion component in the pathogenesis of diarrhea. Rotavirus induces a moderate net chloride secretion at the onset of diarrhea, but the mechanisms appear to be quite different from those used by bacterial enterotoxins that cause pure secretory diarrhea. Rotavirus failed to stimulate Cl^- ^secretion in crypt, whereas it stimulated Cl^- ^reabsorption in villi, questioning, therefore, the origin of net Cl^- ^secretion. A solution to this riddle was that intestinal villi do in fact secrete chloride as a result of rotavirus infection. Also, the overall chloride secretory response is regulated by a phospholipase C-dependent calcium signaling pathway induced by NSP4. However, the overall response is weak, suggesting that NSP4 may exert both secretory and subsequent anti-secretory actions, as did carbachol, hence limiting Cl^- ^secretion. All these characteristics provide the means to make the necessary functional distinction between viral NSP4 and bacterial enterotoxins.

## Background

Viral diarrheas are the cause of high mortality among children and animals, including many mammalian and avian species [1]. But despite considerable research over several decades, the mechanisms underlying rotaviral diarrheal disease remain unclear compared with those of bacterial secretory enterotoxins, such as cholera toxin and the *Escherichia coli *heat-labile and heat-stable toxins.

Rotavirus infection has always been considered to be confined to the upper two-thirds of the villi of the small intestine, but recent reports suggest that extra-intestinal manifestations may occur [2]. Diarrhea can occur with no visible tissue damage and, conversely, the histological lesions can be asymptomatic. The severity of intestinal histological lesions is clearly dependent on both host and viral factors. However, even when slight erosion of the epithelial surface was found to exist in various animal models, there was no evidence of any significant enterocyte loss or flattening of the mucosa [3]. Thus, the idea is gaining ground that diarrhea is not necessarily a consequence of any physical lesion but can precede it, as if cell dysfunction were the cause, not the consequence, of the histological damage [2,4].

Rotavirus diarrhea was first considered to be malabsorptive. Since 1996, the prevailing idea in the rotavirus field is that the nonstructural NSP4 protein might play a crucial role in fluid and electrolyte secretion, and hence might represent a novel viral secretory enterotoxin [5]. Being absent from the mature, infective virion particle, NSP4 needs to be synthesized in virus-infected villus enterocytes and the NSP4 cleavage product NSP4-(112–175) has been reported to be secreted into the extracellular medium and/or the intestinal lumen [6]. Like the full-length NSP4 and NSP4-(114–135) peptide [7], the NSP4-(112–175) peptide induced diarrhea in neonatal mice in the absence of any histological damage to the intestinal mucosa [6]. It has been hypothesized that the NSP4-(112–175) secreted peptide would be available to bind a yet-unidentified apical membrane receptor in villus and perhaps crypt enterocytes and enteroendocrine cells, to trigger a series of events that would lead to secretory diarrhea [2,4,8-11]. Recent studies also indicate that NSP4 is released from the basal side of infected enterocytes, but its role in rotavirus disease remains to be defined [12].

In view of current ideas on the subject of rotavirus and NSP4-mediated diarrhea, we feel that a brief but precise description of the pathological mechanisms involved in the disease is necessary at this time. We further discuss how the mechanisms used by the NSP4 enterotoxin produced by rotavirus appear to be quite different from those used by bacterial enterotoxins in leading to mixed type rather than secretory diarrhea. The aim of this review is to clarify the necessary functional distinction between viral NSP4 and bacterial enterotoxins.

## Current state of knowledge on the mechanisms leading to diarrheal disease

**Figure 1:**
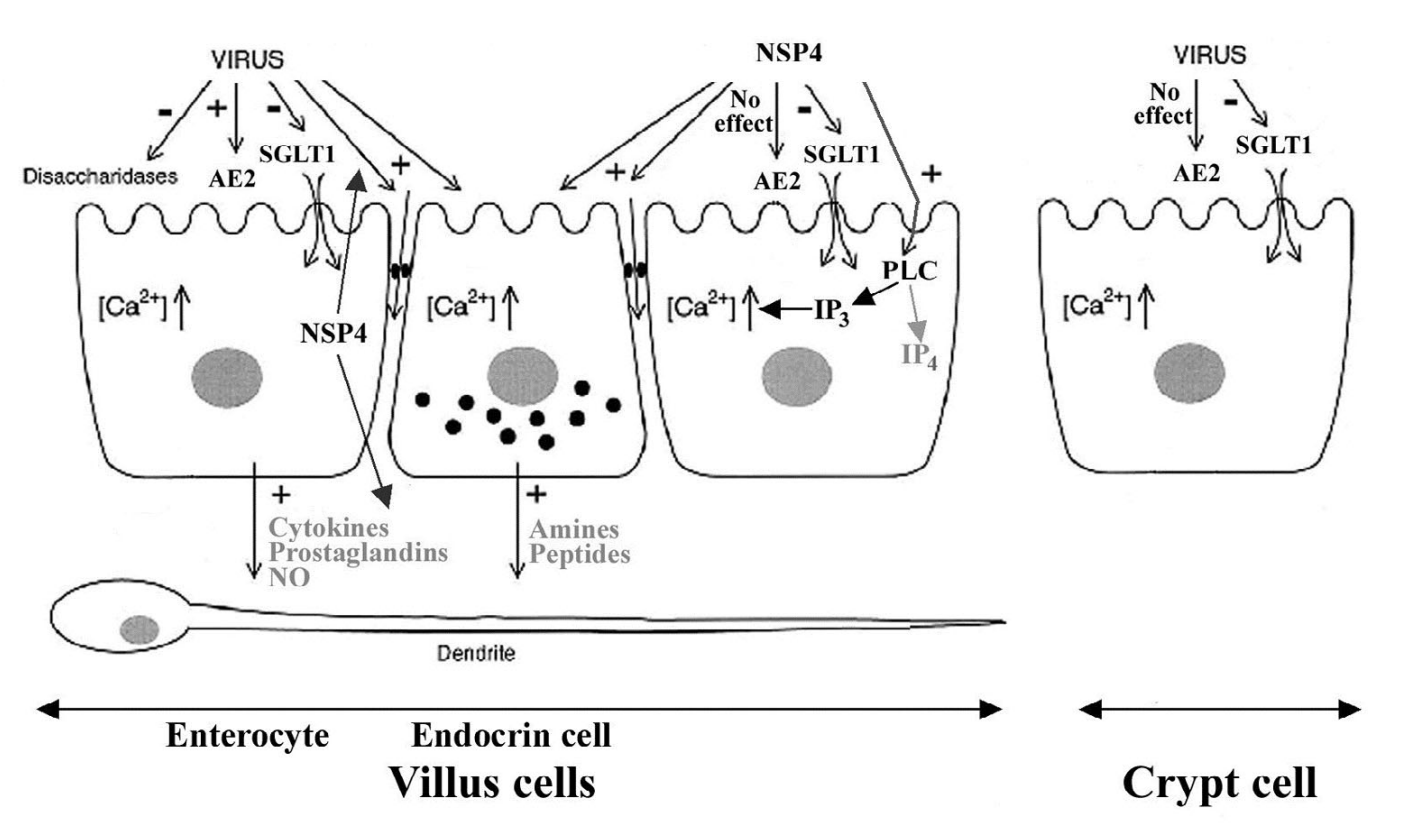
**Reality and hypothesis of the pathophysiological mechanisms of rotavirus and NSP4-mediated diarrhea**. Rotavirus impairs activities of intestinal disaccharidases and Na+-solute symports coupled with water transport, contributing to massive loss of water into the intestinal lumen. NSP4 specifically inhibits SGLT1-mediated Na+-D-glucose symport activity. Rotavirus and/or NSP4 increases epithelial paracellular permeability, but the importance of this effect on fluid and electrolyte fluxes is difficult to evaluate. Loss of Cl^- ^into the intestinal lumen is established by Cl^-^/H^+ ^symport activity (AE2) causing Cl^-^reabsorption or Cl^- ^secretion in villi (depending on the direction of the chloride electrochemical gradient resulting from rotavirus infection), but failing to stimulate Cl^-^transport in crypt. NSP4 has no direct, specific effect on either intestinal absorption or secretion of chloride. Net chloride secretion is regulated by a phospholipase C (PLC)-dependent calcium signaling pathway induced by NSP4. NSP4 is also secreted at the basal side of enterocytes, but its role in rotavirus disease remains to be defined. It has been proposed that NSP4 may exert both secretory and subsequent anti-secretory actions via IP_3 _and IP_4 _respectively, as did carbachol, hence limiting Cl^- ^secretion. It has also been proposed that Ca^2+ ^mobilization may trigger the release of different mediators (cytokines, amines...) which activate the nervous system in the intestinal wall, thereby stimulating Cl^- ^secretion. The signs - and + indicate inhibition and activation, respectively. Further details in the text.

In figure 1, the pathophysiological model of rotavirus-induced diarrhea, adapted from Lundgren and Svensson [4], summarizes the most important effects of rotavirus and NSP4 on the intestinal epithelium.

### Maldigestion and malabsorption of nutrients

It has previously been hypothesized that rotavirus infection kills off most of the mature enterocytes, so that crypt cells invade the villus surface, causing a decrease in the digestive and absorptive capacities of the intestine, and hence generating a malabsorption type of diarrhea [13]. This crypt-cell invasion hypothesis, however, has never been conclusively demonstrated, and in fact has been challenged by more recent work from various laboratories [3,14,15]. A rotavirus-induced decrease in intestinal disaccharidase activities *in vivo *in young mice occurred with relatively intact intestinal brush border membrane (BBM) [15]. Both the activity and expression of sucrase-isomaltase *in vitro *in human Caco-2 cells were reduced by infection without any apparent enterocyte destruction, likely caused by a blockade of protein trafficking to BBM [14]. The SGLT1-mediated Na^+^-D-glucose symport activity present in both villus and crypt cell BBM of rabbit intestine, although higher for villi than for crypt cells, was inhibited by rotavirus in the absence of tissue damage. The glucose uptake value remained much higher for villi BBM in infected rabbits than for crypt cell BBM in control, non-infected rabbits, which would have been improbable if the enterocytes were crypt-like cells [16]. Also, the inhibitions were unaccompanied by any effect on SGLT1 protein expression, which was again higher for villi than for crypt cells, strongly arguing against the crypt-cell invasion hypothesis [3,16]. Because SGLT1 supports water reabsorption under physiological conditions, the mechanism of rotavirus diarrhea may involve a generalized inhibition of Na+-solute symport systems, and hence of water reabsorption. In rabbit intestinal BBM, the NSP4-(114–135) peptide has also been shown to directly and practically instantaneously inhibit SGLT1, but not Na^+^-L-leucine symport activities [17] (Fig. 1). Hence, NSP4 is at least one among other effectors directly causing glucose malabsorption during rotavirus infection *in vivo *[3].

To summarize, rotavirus infection induces maldigestion of carbohydrates and their accumulation in the intestinal lumen as well as malabsorption of nutrients and a concomitant inhibition of water reabsorption, which can lead to a malabsorption component of diarrhea.

### NSP4 enterotoxin

Since the discovery of the NSP4 enterotoxin, diverse hypotheses have been proposed in favor of an additional secretory component in the pathogenesis of rotavirus diarrhea. Hence, there might be common mechanisms of pathogenesis for bacterial and viral enterotoxins [2,4,5,11,18].

In reality, direct experimental evidence of chloride secretion is highly lacking. There is only one instance in the literature in which NSP4 has been shown to directly stimulate iodine influx into distal colon crypts isolated from neonatal mice [19]. The experiments were performed *ex-vivo *on colon crypts, but nothing is known about the direct action of NSP4 on small intestine crypts. Furthermore, the situation *in vivo *is different. Rotavirus infects the mature enterocytes in the upper two-thirds of the villi of the small intestine, and the question arises – as with most luminal enterotoxins – as to the physical accessibility and binding capacity of the secreted NSP4 to the cells of the crypt region [4,18]. On the other hand, the NSP4-(114–135) peptide has been shown to have no direct, specific effect on either intestinal absorption or secretion of chloride [20].

Another possibility is that NSP4 indirectly stimulates chloride secretion. In neonatal mouse intestinal mucosal sheets, NSP4 was initially shown to potentiate cAMP-dependent Cl^- ^secretion [7]. The Cl^- ^secretory currents induced by NSP4-(114–135) were small compared with those induced by cAMP-mobilizing forskolin, which prompted the same authors soon afterwards to publish findings that NSP4, in contrast to many bacterial enterotoxins, led to a cyclic-nucleotide-independent secretory diarrhea [5]. The observation that addition of either NSP4 or carbachol (a cholinergic agonist that mobilizes Ca^2+^) to intestinal mucosal sheets induced transient, small and almost identical increases in Cl^- ^secretory currents was interpreted as indicating that NSP4 induced a Ca^2+^-dependent Cl^- ^secretory mechanism. Evidence has been gathered in favor of the mobilization of intracellular calcium associated with NSP4 expressed endogenously or added exogenously [21-24]. Increasing intracellular calcium is known to induce a small, transient chloride secretion [25-27]. This response contrasts with secretory diarrhea in which activation of the cAMP- or cGMP-dependent intracellular second messenger pathways results in the sustained opening of the apical membrane channels leading to massive Cl^- ^secretion [25-28].

By analogy to bacterial enterotoxin-induced fluid secretion, it has also been proposed that NSP4-mediated Ca^2+ ^mobilization may trigger the release of amines/peptides from intestinal endocrine cells as well as the release of cytokines, prostaglandins and nitrous oxide from the enterocytes [4,29] (Fig. 1). The release of NSP4 to the basal pole of enterocytes has recently been reported in *in vitro *and *in vivo *studies [12]. All these secreted compounds may, alone or together, activate the nervous system (ENS) in the intestinal wall, and hence stimulate intestinal chloride secretion. While ENS involvement may explain how enterotoxins, which most likely do not reach the crypt region, can influence the secreting cells, many details regarding the ENS-linked hypothesis of rotavirus-induced secretory diarrhea remain to be elucidated.

NSP4 has also been found to cause a disruption of tight junctions and a reduction in transepithelial electrical resistance accompanied by an increase in paracellular permeability to macromolecules of 20 kiloDa across MDCK cells [30], as did rotavirus infection in Caco-2 cells [31]. However, the effect of increased paracellular permeability on fluid and electrolyte fluxes remains difficult to evaluate during rotavirus diarrhea *in vivo*.

Taken together, the data show that while NSP4 induces diarrhea in young mice, its importance in chloride secretion remains unresolved. The lack of clear evidence that mobilization of intracellular Ca^2+ ^and subsequent ENS stimulation have a role in indirectly regulating chloride secretion might cast doubts as to the secretory agonist action of NSP4. However, the possibility exists that NSP4 might exert positive as well as negative regulation of Cl^- ^secretion, as did carbachol [25,28,32]. First, Ball et al. reported that, after NSP4 pretreatment, addition of carbachol to intestinal mucosa had no additional effect on Cl^- ^secretory currents [7]. Such a result might be interpreted as a first calcium-dependent Cl^-^secretion that makes the cells refractory to re-stimulation by a second calcium-dependent agonist [7,28]. If NSP4 really exerted an inhibitory effect, its subsequent addition to NSP4 (or carbachol) pretreatment had to give the same negative response, but the authors did not perform such experiments. Second, NSP4 synthesized in rotavirus-infected Caco-2 cells [21] and NSP4 exogenously applied to Spodoptera frugiperda (Sf9) insect cells [23] had been shown to increase intracellular Ca^2+ ^through phospholipase C (PLC) activation. Such PLC activation can lead to transient chloride secretion through inositol (1, 4, 5) triphosphate (IP3) release. However, NSP4, like carbachol, could promote long-term inhibitory feedback through inositol tetrakisphosphate (IP4) production, preventing Cl^- ^secretion from being sustained [28,32]. Hence, the overall chloride secretory response will be determined by the imbalance between the stimulatory and inhibitory effects of NSP4 on the intestinal epithelium. Whether or not the ability of NSP4 to act as a braking mechanism to prevent excessive luminal ion and fluid loss is mediated by IP4 elevation is open to question, but it may account for the moderate net Cl^- ^secretion in rotavirus diarrhea.

### Net chloride secretion

The few available data on intestinal Cl^- ^secretion during rotavirus diarrhea have revealed discordance between *in vitro *studies in Ussing short-circuited chambers and *in vivo *perfusion studies (see [4]). Davidson et al. reported that net Cl^- ^fluxes, like net Na+ fluxes, in jejunal epithelium from piglets infected with human rotavirus were secretory but did not significantly differ from those in non-infected animals [13]. Conversely, Starkey et al. found that when using the perfusion technique Cl^- ^transport exhibited only net Cl^- ^secretion in intestinal segments from rotavirus-infected mice at 72 hours post infection, a time coinciding with both the increase in luminal Cl^- ^concentrations and the peak of diarrheal severity. The authors predicted that net Cl^- ^secretion could not be due to reduced chloride absorption but rather to the presence of a secretory component, but they did not provide any experimental evidence for this suggestion [33]. Lorrot et al. also reported that rotavirus increased luminal Cl^- ^concentrations at 7 days post infection, a time coinciding with the appearance of mild diarrhea in young rabbits [34]. However, the increase in luminal Cl^- ^concentrations was found to be moderate, which would seem to be in line with observations that the ionic concentrations in the stools of rotavirus-positive children are much lower than those found in the pure secretory diarrheas caused by secretagogues such as the enterotoxins of *Vibrio cholerae *and *Escherichia coli*.

While it is widely accepted that loss of Cl^- ^in the stools can be due to decreased absorption in the villus cells and/or increased secretion in the crypt cells [25,35-37], the mechanisms of rotavirus-mediated diarrhea appear to be rather different. Recently, Lorrot et al. reported that rotavirus infection in young rabbits failed to stimulate Cl^- ^transport in crypt cell BBM [16], whereas it stimulated Cl^- ^reabsorption in villus cell BBM [34] (Fig. 1). As regards the overall chloride secretory response, these mechanisms appeared unable to explain the moderate increase in net Cl^- ^secretion at the onset of rotavirus diarrhea. Because rotavirus stimulated both Cl^- ^influx and Cl^- ^efflux in villi, Lorrot et al. proposed that the chloride carrier might function in both normal (absorption) and reversed (secretion) modes, depending on the direction of the chloride electrochemical gradient resulting from rotavirus infection [16]. The presence of the Cl^-^/H^+ ^symporter may also explain why rotavirus still results in diarrhea in mice lacking the apical CFTR (Cystic Fibrosis Transmembrane conductance Regulator) chloride channel [19].

## Mode of action of the viral and bacterial enterotoxins

The mechanisms by which rotavirus and the NSP4 enterotoxin cause diarrhea appear to be quite different from those described for bacterial enterotoxins, such as cholera toxin and the heat-labile and heat-stable enterotoxins of *Escherichia coli*, which cause "pure" secretory diarrhea [36]. To support this proposal, the main characteristics of diarrheal diseases associated with bacterial and NSP4 viral enterotoxins are given in Table 1. It appears that the only common characteristic of bacterial and viral enterotoxins is that neither causes morphological damage [7,11,36]. Bacterial enterotoxins are known to have no effect on coupled absorption of Na^+ ^and glucose [36]. On the other hand, rotavirus has been shown to impair Na^+^-solute symport activities, hence contributing to massive water loss all along the small intestinal crypt-villus axis [3]. NSP4 was also able to directly and specifically inhibit SGLT1 [17]. Unlike enterotoxigenic diarrhea in which chloride malabsorption is coupled with chloride hypersecretion, leading to the massive loss of Cl^- ^[36,38], rotavirus was found to cause substantial Cl^- ^reabsorption in villi [34] without stimulating Cl^- ^transport in crypt [16], questioning, therefore, the origin of net Cl^- ^secretion. A solution to this riddle was that intestinal villi do in fact secrete chloride as a result of rotavirus infection [16]. NSP4 has no direct, specific effect on either intestinal absorption or secretion of chloride [20]. Finally, the intracellular mediators of chloride secretion were also different (Table 1). In contrast to the sustained secretory responses induced by cyclic nucleotides, the Ca^2+^-dependent chloride secretory response induced by rotavirus is transient and small, implying that negative signaling events may limit chloride secretion [25]. The possibility that NSP4 may be able to exert both secretory and subsequent anti-secretory actions, as did carbachol, remains to be verified experimentally, but may explain the moderate loss of Cl^-^into the intestinal lumen at the onset of rotavirus diarrhea. All these considerations support the idea that NSP4 may act as an enterotoxin, but it would act as a viral enterotoxin that would function quite differently from bacterial enterotoxins by inducing mixed type rather than secretory diarrhea.

**Table 1:** Major characteristics of diarrheal diseases associated with bacterial and NSP4 viral enterotoxins.

	**Bacterial enterotoxins: *Vibrio cholerae and Escherichia coli***	**Viral enterotoxin: NSP4 produced by rotavirus**
Morphological damage	No	No
D-glucose absorption via SGLT1	Unimpaired	Impaired
Cl^- ^absorption at the villus cell level	Decreased	Increased
Cl^- ^secretion at the crypt cell level	Increased	Unaffected
Net Cl^- ^secretion	Massive	Moderate
Intracellular mediators of chloride secretion	Cyclic nucleotides (cAMP or cGMP)	Ca^2+^

Bacterial enterotoxins such as cholera toxin and the heat-labile and heat-stable enterotoxins of *Escherichia coli *are known to cause "pure" secretory diarrhea [36]. The NSP4 viral enterotoxin produced by rotavirus appears to function quite differently from bacterial enterotoxins, aside from the fact that neither type causes morphological damage. Further details in the text.
